# Supplementary material for: Development of a national quality framework for palliative care in a mixed generalist and specialist care model: A whole-sector approach and a modified Delphi technique
Source: PLoS One. 2022 Mar 23;17(3):e0265726. doi: 10.1371/journal.pone.0265726 (PMC8942240; doi:10.1371/journal.pone.0265726)
Supplement: S1 Appendix — (PDF) [file pone.0265726.s001.pdf]

Supporting information 1

Stakeholder associations and organisations involved in whole-sector development of the Netherlands Quality Framework for Palliative Care

STEERING GROUP
Branch association for nursing homes, home care, rehabilitation care and youth welfare services (Actiz)
Netherlands Comprehensive Cancer Organisation (IKNL)
Royal Dutch Medical Association (KNMG)
National Association of General Practitioners (LHV)
Dutch College of General Practitioners (NHG)
Netherlands Federation of University Medical Centres (NFU)
Netherlands Patient Federation (PFNL)
Netherlands Association of Physicians for the Intellectually Disabled (NVAVG)
Netherlands Association for Psychosocial Oncology (NVPO)
Dutch Hospital Association (NVZ)
Dutch Society of Professionals in Palliative Care (Palliactief)
Quality of Care Council – Dutch Federation of Medical Specialists (RK-FMS)
Dutch Federation of Oncological Societies (SONCOS)
Netherlands Association for Nurses and Carers (V&VN)
Dutch Association of Elderly Care Physicians (Verenso)
Dutch Association of Spiritual Caregivers (VGVZ)
Netherlands umbrella organization of health insurers (ZN)

SOUNDING BOARD
Centre for Social and Spiritual Support in Palliative Care (Agora)
Netherlands Association for Hospice Care (AHzN)
Palliative Care Expertise Centres (EPZ)
Royal Dutch Society for Physical Therapy (KNGF)
Dutch Cancer Society (KWF)
Netherlands Institute for Health Services Research (NIVEL)
National Programme for Palliative Care (NPPZ)
Cooperation of General Hospitals (SAZ)

SOUNDING BOARD (continued)
Dutch Platform for Palliative Care Networks (Stichting Fibula)
PAL Foundation for Pediatric Expertise in Palliative Care (Stichting PAL)
Cooperation of Top-Clinical Care Hospitals (STZ)
Volunteers for Palliative Care at the End of Life (VPTZ)
Ministry of Health, Welfare and Sport (Ministerie van VWS)
National Health Care Institute (ZiN)
Netherlands Organisation for Health Research and Development (ZonMw)

EXPERT PANEL
Dutch Federation of Medical Specialists (FMS)
Dutch College of General Practitioners (NHG)
Netherlands Patient Federation (PFNL)
Netherlands Association for Psychosocial Oncology (NVPO)
Netherlands Association for Nurses and Carers (V&VN)
Dutch Association of Elderly Care Physicians (Verenso)
Dutch Association of Spiritual Caregivers (VGVZ)
Netherlands umbrella organization of health insurers (ZN)

OTHER INTERESTED PARTIES IN THE ROUND-TABLE DISCUSSIONS
Health and Youth Care Inspectorate (IGJ)
Dutch Cancer Society (KWF)
Dutch Federation of Cancer Patient Organisations (NFK)
Dutch Association for Anaesthesiology (NVA)
Dutch Association for Paediatric Medicine (NVK)
Standard for Palliative Care 1.0 Development Group
Project Group for Quality Indicators
Project Group for Palliative Care in District Nursing
