# Supplementary material for: Development of a national quality framework for palliative care in a mixed generalist and specialist care model: A whole-sector approach and a modified Delphi technique
Source: PLoS One. 2022 Mar 23;17(3):e0265726. doi: 10.1371/journal.pone.0265726 (PMC8942240; doi:10.1371/journal.pone.0265726)
Supplement: S2 Appendix — (PDF) [file pone.0265726.s002.pdf]

Supporting Information 2

Delphi survey for the End-of-Life Care domain in the Netherlands Quality Framework for Palliative Care

In this document* please find:

1. An overview of the core documents selected for construction of the Netherlands Quality Framework for Palliative Care (NQFPC)
2. A glossary of relevant terms.
3. A table containing all standards related to the End-of-Life Care domain from the various core documents accompanied by a clarification of the procedure to select relevant and suitable standards for the NQFPC.
4. Tables containing all criteria related to the End-of-Life Care domain from the various core documents accompanied by a clarification of the procedure to select relevant and suitable criteria for the NQFPC.
5. Reply sheet

*Similar documents for other domains of the NQFPC are available on request.

1. Core documents

Below you find an overview of the documents selected as core documents for constructing the NQFPC. These documents resulted from a literature search performed by the project team of peers and endorsed by the working group (see figure 2 in manuscript). Below the full name of the documents you will find the abbreviation used for identification by the project team in the Delphi procedure.

- Standards for providing quality palliative care for all Australians, PCA (2005)
  (AU)
- Hospice New Zealand standards for palliative care, Hospice NZ (2012)
  (NZ)
- Clinical practice guidelines for quality palliative care, NCP (2013)
  (NCP)
- Quality standard end of life care for adults, NICE (2013)
  (NICE)
- Standard for Palliative Care 1.0, CBO (2013)
  (ZM)
- Prezo Audit for Hospicecare, Perspekt (2015)
  (PREZO)

2. Glossary of relevant terms

The NQFPC consists of several domains that together cover all aspects of palliative care.
Domain: a demarcated area that describes a specific dimension of palliative care.
Standard: indicates best practice within a domain, taking into account recent findings and evidence.
Criterion: a condition which a specific aspect of care must meet to obtain the desired standard.
End of life care: care in the last days (up to 7 days) of life.

3. Standards

Clarification of procedure

On the next page the standards related to the End of Life Care domain in the various core documents are displayed in a table. The project team made an effort to group comparable standards as much as possible and aligned them horizontally. The column on the far right contains the standards proposed by the project team as suitable for the NQFPC.

N.B.

The Standard for Palliative Care 1.0, CBO (2013) describes criteria and no standards. Therefore, there is no column referring to the Standard for Palliative Care in this table.

Request to the working group

On the reply sheet at the end of this document members of the working group are kindly requested to indicate whether they can agree with the proposed standards or which other standards they would consider better suitable for the NQFPC. The numbers of the standards in the table below correspond to the numbers referred to on the reply sheet.

Table of standards relevant to the End of Life Care domain

AU(2005)	NZ(2012)	NCP (2013)	NICE (2013)	Prezo (2015)	Proposed standard for NQFPC
The unique needs of dying patients are considered, their comfort maximized and their dignity preserved. (AU_6)	The unique needs of patients in the last days of life, and the family and whanau, are considered, their comfort maximized and their dignity preserved. (NZ_8)	The interdisciplinary team (IDT) identifies, communicates, and manages the signs and symptoms of patients at the end of life to meet the physical, psychosocial, spiritual, social, and cultural needs of patients and families. (NCP_7.1)	People in the last days of life are identified in a timely way and have their care coordinated and delivered in accordance with their personalised care plan, including rapid access to holistic support, equipment and administration of medication. (NICE_Statement 11)	De patiënt ervaart aandacht, comfort en ondersteunende zorg bij het afronden van het leven. (PREZO_Domein 6)	People in the last days of life are identified in a timely way and have their care coordinated and delivered in accordance with their personalised care plan, including rapid access to holistic support, equipment and administration of medication. (NICE_Statement 11)
		The IDT assesses and, in collaboration with the patient and family, develops, documents, and implements a care plan to address preventative and immediate treatment of actual or potential symptoms, patient and family preferences for site of care, attendance of family and/or community members at the bedside, and desire for other treatments and procedures. (NCP_7.2)			The IDT assesses and, in collaboration with the patient and family, develops, documents, and implements a care plan to address preventative and immediate treatment of actual or potential symptoms, patient and family preferences for site of care, attendance of family and/or community members at the bedside, and desire for other treatments and procedures. .(NCP_7.2)
		Respectful postdeath care is delivered in a respectful manner that honors the patient and family culture and religious practices. (NCP_7.3)	The body of a person who has died is cared for in a culturally sensitive and dignified manner. (NICE_Statement 12)		Respectful postdeath care is delivered in a respectful manner that honors the patient and family culture and religious practices. (NCP_7.3)
			Families and carers of people who have died receive timely verification and certification of the death. (NICE_Statement 13)		
		An immediate bereavement plan is activated postdeath. (NCP_7.4)	People closely affected by a death are communicated with in a sensitive way and are offered immediate and ongoing bereavement, emotional and spiritual support appropriate to their needs and preferences. (NICE_Statement 14)		An immediate bereavement plan is activated postdeath. (NCP_7.4)

4. Criteria

Clarification of procedure

On the next pages all criteria related to the standards of the End of Life Care domain in the various core documents are displayed in several tables. To facilitate the working group, the project team made an effort to group criteria as much as possible referring to specific phases in End of Life Care. The tables have the following subjects regarding end of life care:

- General
- Pre-death
- Peri-death
- Post-death
- Other

N.B.

NICE (2011) describes standards (statements) without additional criteria. It does describe accessory indicators, however these do not fit the scope of the NQFPC. Therefore, there is no column referring to NICE (2011) in the following tables.

Request to the working group

On the reply sheet at the end of this document members of the working group are kindly requested to indicate for each criterion whether it should be incorporated in the NQFPC. Each criterion carries a unique combination of letters and numbers. These combination – codes can be found in the far left column on the reply sheet.

Table of general criteria relevant to End of Life Care domain

Criteria AU (2005)	NZ (2012)	Criteria NCP (2013)	Criteria Zorgmodule (2013)	Criteria Prezo (2015)
Guidance and support is given to primary care providers regarding decision making and end of life care (AU_6.9)	-	Care of the patient at the end of life is time and detail intensive, requiring expert clinical, social, and spiritual attention to the process as it evolves. Care of the patient is divided into three phases; pre death, peri death, and post death. (NCP_7.1.1)	-	De patiënt of zijn naaste geeft aan en/of heeft aangegeven wat hij comfortabel vindt om zijn lichamelijke, psychische, sociale en spirituele klachten en symptomen zoveel mogelijk te verminderen. (PREZO_domein6.1p)
Protocols to guide care at the end of life are developed and disseminated (AU_6.10)				de medewerker zorgt voor zoveel mogelijk comfort voor de patiënt (PREZO_domein6.1m)
Processes are established to respond to the need for urgent assessment and guidance (AU_6.11)				de medewerker bespreekt verlies- en rouwgevoelens met de patient en/of naasten als zij hiervoor open staan (PREZO_domein6.3m)
Guidance and support is available to health care providers seeking advice about ethical dilemmas related to end of life care and decision-making. (AU_6.12)				de medewerker heeft aandacht voor emoties bij de patient en/of naaste (PREZO_domein6.4m)
				De medewerker stelt de draagkracht en draaglast van de patient en zijn naasten vast, signaleert mogelijke overbelasting en handelt hiernaar (PREZO_domein6.7m)
				De medewerker legt de gemaakte afspraken in het individueel zorgplan vast, evalueert deze met de patient en stelt zo nodig de afspraken bij (PREZO_domein6.8m)

Table of criteria relevant to pre-death phase in End of Life Care domain

Criteria AU (2005)	Criteria NZ (2012)	Criteria NCP (2013)	Criteria Zorgmodule (2013)	Criteria Prezo (2015)
Regular and ongoing assessment of the patient identifies transition into the terminal phase. (AU_6.1)	the concerns, hopes, fears and expectations of the imminently dying patient and their family and whanau are discussed openly and honestly in a way that is appropriate for their age, culture, spiritual and social situation. (NZ_8.1)	With the patient and family, a plan is developed to meet their unique needs during the dying process as well as the needs of family immediately following the patient's death. Reassessment and revision of the plan occurs in a timely basis. (NCP_7.2.2)	Monitor het stervensproces en pas de zorg daarop aan (ZM_stervensfase_4.1)	De patiënt of zijn naaste geeft aan en/of heeft aangegeven wat voor hem belangrijk is bij de afronding van het leven zoals:<br>-aandacht voor de betekenis die hij geeft aan kwaliteit van leven en sterven<br>-het leven voltooien in waardigheid en eigenheid<br>-emotionele steun<br>-begeleiding bij beslissingen rondom het levenseinde<br>-aandacht voor waarden en keuzen<br>-wensen en behoeften op lichamelijk, psychisch, sociaal en spiritueel gebied<br>-uitvoeren van rituelen<br>-steun bij het nemen van afscheid en loslaten van het leven<br>-betrokkenheid en ondersteuning van naasten bij en na het overlijden (PREZO_domein6.2p)
End of life issues and anticipation of death are honestly discussed with the patient, their caregiver/s and family in a socially and culturally appropriate manner. (AU_6.2)	the family is educated regarding the signs and symptoms of approaching death, in a way that is appropriate for their age, culture and social situation. (NZ_8.3)	In collaboration with the patient and family, the IDT provides care with respect for patient and family values, preferences, beliefs, culture, and religion. (NCP_7.1.4)	Stel vast wie nazorg geeft en dient te ontvangen (ZM_stervensfase_5.2)	Patiënt of zijn naaste bepaalt en/of heeft aangegeven welke activiteiten in zijn stervensfase:<br>-hij zelf wil uitvoeren<br>-door zijn naasten uitgevoerd worden<br>-door vrijwilligers/medewerkers/organisatie uitgevoerd kunnen worden. (PREZO_domein6.3p)
Symptoms at the end of life are assessed and documented with appropriate frequency and treatment and care is based on patient, their caregiver/s and family needs and wishes. (AU_6.3)	information and discussion on after death wishes and decisions is facilitated, if wanted (NZ_8.4)	Before the patient's death, sensitive communication occurs, as appropriate, about autopsy, organ and tissue donation, and anatomical gifts, adhering to institutional and regional policies. (NCP_7.2.5)		de medewerker inventariseert met de patiënt en/of zijn naasten wensen en behoeften t.a.v. het stervensproces en de praktische zaken vlak voor en na het overlijden en zet zich in om deze te realiseren (PREZO_domein6.2m)
The caregiver/s and family members are given information		Any inability to honor the patient's and family's expressed wishes for care		de medeweker signaleert, inventariseert en beoordeelt, zo mogelijk met behulp van

regarding the signs and symptoms of approaching death in a manner appropriate to their individual needs and circumstances. (AU_6.4)		immediately leading up to and following the patient's death is documented and communicated in the medical record that is accessible to other health care providers. (NCP_7.2.3)		instrumenten, de aard en ernst van de klachten, risico's en/of symptomen die de patient ervaart (fysiek: pijn, benauwdheid, misselijkheid/braken; emotioneel: angst, depressiviteit, boosheid, ontremming; cognitief: moeite met denken, begrijpen, onthouden, plannen & organiseren, besef van tijd, plaats of persoon). Bespreekt dit met patient en/of naasten en neemt in overleg passende en preventieve maatregelen (PREZO_domein6.5m)
		For patients who have not accessed hospice services, care planning at this stage may include the introduction or reintroduction of hospice referral, if such an option is congruent with the patient's and family's goals and preferences. (NCP_7.2.4)		De medewerker signaleert en inventariseert samen met de patient (pro)actief:<br>-situaties waarin volstaat kan worden met alledaagse aandacht voor het afronden van het leven<br>-situaties waarin de patient behoefte heeft aan begeleiding bij het afronden van het leven en situaties waarbij begeleiding door een deskundige toegevoegde waarde kan hebben<br>-situaties waarin een crisis ontstaat die vraagt om een crisisinterventie door een expert<br>(PREZO_domein6.9m)

Table of criteria relevant to peri-death phase in End of Life Care domain

Criteria AU (2005)	Criteria NZ (2012)	Criteria NCP (2013)	Criteria Zorgmodule (2013)	Criteria Prezo (2015)
Provision is made to enable the patient and family to participate in customary or religious end of life rituals that have meaning for them. (AU_6.5)	When the patient is assessed as imminently dying the care plan is revised to reflect the needs of the patient and family and a last days of life care pathway is implemented. (NZ_8.2)	The IDT assesses the patient for symptoms and proactively prepares family and other caregivers on the recognition and management of potential symptoms and concerns. (NCP_7.2.1)	Vaststellen van het stervensproces en monitoren van het zorgplan (ZM_stervensfase_3.1)<br><br>Besteed aandacht aan afscheid en rouw bij patiënt en naasten (ZM_stervensfase_ 1.4)	de medewerker gaat in de stervensfase na:<br>-wat de patient zelf wil doen<br>-wat de naasten van de patient doen<br>-wat de vrijwilligers/medewerkers/organisatie doen (PREZO_domein6.6m)
Plans are in place for the certification of death should this occur out of hours. (AU_6.7)	There are processes in place to respond to the need for urgent assessment and guidance for all providers caring for patients in the last days of life . (NZ_8.6)		Vraag de stervende naar storende symptomen (ZM_stervensfase_ 1.2)<br><br>Let op signalen die wijzen op (naderend) sterven (ZM_stervensfase_ 2.1)<br><br>Let op symptomen en pas het onderzoek aan (ZM_stervensfase_ 2.2)<br><br>Vraag naasten naar opvallende signalen en symptomen en exploreer deze (ZM_stervensfase_ 1.3)	
	Guidance and support is available to health care providers seeking advice about ethical dilemmas related to last days of life care and decision making. (NZ_8.7)		Zorg is gericht op comfort (ZM_stervensfase_ 1.1)	

Table of criteria relevant to post-death phase in End of Life Care domain

Criteria AU (2005)	Criteria NZ (2012)	Criteria NCP (2013)	Criteria Zorgmodule (2013)	Criteria Prezo (2015)
Immediately following the death of the patient and during the early bereavement phase, caregiver/s and families are given time and continuing care to provide comfort, and assist with adjustment to the death of the patient. (AU_6.6)	-	In post death, the focus of care includes respectful care of the body and support of the family. (NCP_7.3.1)	Geef ruimte voor rituelen (ZM_stervensfase_ 4.3)	-
All providers of care (in particular the patient's general practitioner) receive information about the end phase of illness and/or death as soon as possible. Personal communication via telephone precedes official written communication. (AU_6.8)		The IDT assesses and documents cultural and religious practices particular to the post death period, and delivers care honoring those practices, in accordance with both institutional practice, local laws, and state regulations next to domain 5: spiritual, religious, and existential aspects of care and domain 6: cultural aspects of care. (NCP_7.3.2)	Realiseren van praktische afspraken (wie verwittigen) (ZM_stervensfase_ 5.1)	
		As described in domain 3: psychological and psychiatric aspect of care the IDT formulates and activates a post death bereavement plan based on a social, cultural, and spiritual grief assessment. (NCP_7.4.1)		
		A health care team member is assigned to support the family in the post death period and assist with religious practices, funeral arrangements, and burial planning. (NCP_7.4.2)		

Table of other criteria relevant to End of Life Care domain

criteria AU (2005)	NZ (2012)	criteria NCP (2013)	Zorgmodule (2013)	Prezo (2015)
-	Plans are in place for the certification of death, including plans for certification after hours.(NZ_8.5)	The IDT recognizes the need for high acuity and high intensity care during the dying process. (NCP_7.1.2)	Geef adviezen over houding en (stoppen met) voeding (ZM_stervensfase_ 4.2)	-
	Support all providers of palliative care to adopt and implement a last days of life care pathway. (NZ_8.8)	The IDT routinely elicits and honestly addresses concerns, hopes, fears, and expectations about the dying process in a developmentally appropriate manner, with respect for the social and cultural context of the family next to domain 6: cultural aspects of care. (NCP_7.1.3)		
		The IDT acknowledge the patient's needs at the end of life and educate the family and other care providers about what to expect in terms of death. As death approaches, they communicate signs and symptoms of imminent death, in culturally and developmentally appropriate language, with attention to population specific issues and age appropriateness. (NCP_7.1.5)		

References

- Palliative Care Australia. Standards for providing Quality Palliative Care for all Australians, 2005.
- National Institute for Health and Care Excellence. Quality standard for end of life care for adults, 2011.
- Hospice New Zealand. Hospice New Zealand Standards for Palliative Care. Wellington, 2012.
- National Consensus Project for Quality Palliative Care. Clinical practice guidelines for quality palliative care. Pittsburgh: National Consensus Project for Quality Palliative Care, 2013.
- CBO. Zorgmodule Palliatieve Zorg 1.0, 2013.
- Stichting Perspekt. Prezo Hospicezorg. Utrecht, 2015.

Replysheet

Domain: End of Life Care

Name working group member: _

Standards

Members of the working group are kindly requested to indicate whether they can agree with the proposed standards or which other standards they would consider better suitable for the NQFPC. The numbers of the standards mentioned in the reply table below correspond to the numbers indicated after each proposed standard in the "table of standards relevant to the End of Life Care domain".

In the table below please indicate for each proposed standard:

- whether you can agree and
- If, and how, you would want to adapt or extend the text of the proposed standard; or
- whether you disagree (please add argumentation)

When you prefer to add another standard from the table or you find there is a standard missing for this specific domain, please indicate this in the bottom table.

**Number of standard**	**Agree, (with potential suggestion for adaptation / extension)**	**Disagree (please add argumentation)**
NICE_Statement 11		
NCP_7.2		
NCP_7.3		
NCP_7.4		

**Suggestions for additional standards for this specific domain**:

Criteria

Members of the working group are kindly requested to indicate for each criterion mentioned in the five separate tables above whether it should be incorporated in the NQFPC. Each criterion carries a unique combination of letters and numbers. The numbers of the criteria mentioned in the reply table below correspond to these combination – codes indicated after each criterion in the five tables above.

In the table below please indicate for each criterion:
- whether the criterion should be incorporated and
- If, and how, you would want to adapt or extend the text of the criterion; or
- whether the criterion should not be incorporated (please add argumentation)

When you find there is a criterion missing for this specific domain, please indicate this in the bottom table.

Examples of reasons not to incorporate a criterion are:
- the criterion is not suitable or appropriate for this specific domain
- the criterion is more suitable for the primary domain "Core values and principles".
- the criterion is similar to another criterion that you already indicated to incorporate.
- the content of the criterion is not suitable for the Dutch healthcare setting

The selected and newly suggested standards and criteria will be presented and discussed in the next working group meeting. After agreement on incorporation, the domain will be constructed for presentation to Steering group, Sounding board group and consultation of peers.

**Number of criterion**	**Yes, incorporate in this domain, (with potential suggestion for adaptation / extension)**	**No, do not incorporate (please add argumentation)**
General		
AU_6.9		
AU_6.10		
AU_6.11		
AU_6.12		
NCP_7.1.1		
PREZO_domein6.1p		
PREZO_domein6.1m		
PREZO_domein6.3m		

PREZO_domein6.4m		
PREZO_domein6.7m		
PREZO_domein6.8m		
Pre-death		
AU_6.1		
AU_6.2		
AU_6.3		
AU_6.4		
NZ_8.1		
NZ_8.3		
NZ_8.4		
NCP_7.2.2		
NCP_7.1.4		
NCP_7.2.5		
NCP_7.2.3		
NCP_7.2.4		
ZM_stervensfase_4.1		
ZM_stervensfase_5.2		
PREZO_domein6.2p		
PREZO_domein6.3p		
PREZO_domein6.2m		
PREZO_domein6.5m		
PREZO_domein6.9m		
Peri-death		
AU_6.5		
AU_6.7		
NZ_8.2		
NZ_8.6		
NZ_8.7		
NCP_7.2.1		
ZM_stervensfase_3.1		
ZM_stervensfase_1.4		
ZM_stervensfase_1.2		
ZM_stervensfase_2.1		
ZM_stervensfase_2.2		
ZM_stervensfase_1.3		
ZM_stervensfase_1.1		

PREZO_domein6.6m		
Post-death		
AU_6.6		
AU_6.8		
NCP_7.3.1		
NCP_7.3.2		
NCP_7.4.1		
NCP_7.4.2		
ZM_stervensfase_ 4.3		
ZM_stervensfase_ 5.1		
overige		
NZ_8.5		
NZ_8.8		
NCP_7.1.2		
NCP_7.1.3		
NCP_7.1.5		
ZM_stervensfase_ 4.2		

**Suggestions for additional criteria for this specific domain**:
