# Supplementary material for: Development of a national quality framework for palliative care in a mixed generalist and specialist care model: A whole-sector approach and a modified Delphi technique
Source: PLoS One. 2022 Mar 23;17(3):e0265726. doi: 10.1371/journal.pone.0265726 (PMC8942240; doi:10.1371/journal.pone.0265726)
Supplement: S3 Appendix — (PDF) [file pone.0265726.s003.pdf]

Supporting information 3.

Bottleneck analysis of palliative care provision in the Netherlands

Bottlenecks / Barriers		Literature Inventory (2005 – 2015)	Hospital Survey	Invitational Conference Primary Care
**Information & Communication**	Patients	▪ Flawed information (treatment options, pro's & con's, expected results);<br>▪ Written information is scarce;<br>▪ Unclear communication about prognosis, often (just) shortly before dying;<br>▪ Insufficient involvement in decision making.		
	Healthcare professionals			▪ Insufficient engagement with patients about death and dying and advance care planning.
**Coordination & Continuity of Care**	Patients	▪ Inadequate coordination between healthcare professionals;<br>▪ Inadequate transfer information between hospital and primary care;<br>▪ Difficulty getting a second opinion;<br>▪ Difficulty in reaching healthcare organizations;<br>▪ Irregular visits of community-based nurses;<br>▪ Many different healthcare professionals per patient;<br>▪ Difficulties in handling acute problems (fear for sudden hospital admission).		
	Healthcare professionals	▪ Difficult cooperation with community-based nurses (too many community care organizations);<br>▪ Inadequate information transfer between hospital and primary care;<br>▪ Inadequate or insufficient information transfer to out-of-hours physician services;<br>▪ No clear agreement on 'leading healthcare professional';	▪ No clear assignment of the board for specialist palliative care.<br>▪ Lack of continuity of care through lack of staff.	▪ Insufficient coordination of care with primary care physician while patient is treated by medical specialist;<br>▪ Inadequate information transfer between hospital and primary care;<br>▪ Discontinuity of care out-of-hours;<br>▪ Inadequate or insufficient information transfer to out-of-hours physician services;

		▪ Inadequate coordination between hospital and primary care;<br>▪ Insufficient continuity of care from healthcare professionals;<br>▪ Higher frequency in transfer of care due to increase in part time working healthcare professionals.		▪ Inadequate information transfer between primary care physicians and community based nurses;<br>▪ Primary care physician has no access to digital information system of community care organizations;<br>▪ No clear agreement on 'leading healthcare professional';<br>▪ Through insufficient information transfers, healthcare professionals cannot provide appropriate care to their patients and patients have to frequently repeat their needs or wishes.
**Expertise, training & education**	Patients	▪ Insufficient spiritual care;<br>▪ Insufficient psychosocial support (anxiety, depression);<br>▪ Inadequate support of physical symptoms (dyspnea, pain, constipation);<br>▪ Lack of education and training (especially among primary care physicians).		
	Healthcare professionals	▪ Healthcare professionals have a different understanding of the concept of palliative care;<br>▪ No acknowledgement of the palliative care trajectory;<br>▪ No anticipating the palliative care trajectory;<br>▪ Difficulty in discussing end of life;<br>▪ Lack of advance care planning;<br>▪ Insufficient attention for psychological support;<br>▪ Insufficient attention for spiritual needs;<br>▪ Insufficient knowledge about death and dying in other cultures;<br>▪ Insufficient attention for informal caregiver(s);<br>▪ Family physicians have insufficient knowledge of locally or regionally provided care;<br>▪ Focus on treatment of the disease.	▪ Unconscious incompetence among colleagues;<br>▪ Unfamiliarity with palliative care;<br>▪ Reluctance in identifying palliative care trajectory;<br>▪ Unaware of added value of specialist palliative care<br>▪ There is no room for palliative care in disease-modifying treatment	▪ Lack of knowledge and competence with regard to palliative care;<br>▪ Insufficient advance care planning.

**Rules, Regulations & Reimbursement**	Healthcare professionals	▪ Bureaucracy to obtain palliative care indication;<br>▪ High administration load.	▪ Ambiguity on reimbursement and available finances;<br>▪ Lack of dedicated time / hours.	▪ Palliative care is intensive care with regard to patient care as well as coordination of care with other healthcare professionals. Reimbursement for providing appropriate palliative care is insufficient, fragmented and complex to obtain;<br>▪ Some reimbursements require a statement of limited life expectancy. Aside from the extra administration load, this does not align with the current view that palliative care is best integrated early, i.e. the last year of life.<br>▪ The goal of efficiency in the current healthcare system does not align with the amount of time required to provide appropriate care for patients and their families and to coordinate care with other healthcare professionals.

References

IKNL/Palliactief. Knelpuntenanalyse Palliatieve Zorg in de eerste lijn (Bottleneck analysis of primary palliative care). Utrecht, 2017.

IKNL/Palliactief. Palliatieve Zorg in Nederlandse ziekenhuizen (Palliative care in Dutch hospitals). Utrecht, 2015.

Brinkman-Stoppelenburg A, Boddaert M, Douma J, et al. Palliative care in Dutch hospitals: a rapid increase in the number of expert teams, a limited number of referrals. *BMC Health Serv Res.* 2016;16(1):518.
