# Supplementary material for: Development of a national quality framework for palliative care in a mixed generalist and specialist care model: A whole-sector approach and a modified Delphi technique
Source: PLoS One. 2022 Mar 23;17(3):e0265726. doi: 10.1371/journal.pone.0265726 (PMC8942240; doi:10.1371/journal.pone.0265726)
Supplement: Attachment — Submitted filename: Response to Reviewers.docx [file pone.0265726.s006.docx]

**Title:** Manuscript Submission ID PONE-D-21-32051 entitled
" Development of a national quality framework for palliative care in a mixed generalist and specialist care model: A whole-sector approach and a modified Delphi technique”

Dear Tim Luckett,

We thank you and the reviewers for your valuable comments on our manuscript.

We tried to address all comments of the reviewers in an appropriate way and we believe that the suggestions have improved our manuscript considerably. Below you will find our point by point responses to the reviewers’ comments. We are looking forward to hearing from you.

Sincerely, on behalf of all the co-authors,

Manon Boddaert

**Response to Reviewers**

Reviewer #1:

Thank you for the opportunity to review this interesting paper. It is exceptionally well written and provides a clear and coherent account of the complex process of developing a national quality framework for palliative care in the Netherlands. There is important and potentially transferable information on how to develop such a framework taking an inclusive approach. It is helpful to see that their vision included seeing the framework as including civil society rather than regarding palliative care merely as a medical discipline.

There are a few minor points that would benefit from attention:
1. Page 6 Line 147-148 and Pages 9-10 The authors refer to a search of the palliative care literature. It is not clear if this literature review is already published?

**Author’s reply:**

We appreciate your detailed attention. The review of Dutch literature mentioned on page 6 was conducted for the bottleneck analysis and has not been published separately. It has, however, been included as part of a report of the bottleneck analysis, to which we make a reference in the Results section on page 9-10. Therefore, we have included this reference in the section you mentioned (Methods section, e. Bottleneck analysis, page 6):

*….Therefore, a search of Dutch palliative care literature published between 2005 and* *2015 was performed [1]…..*

2. Page 11-12 There is little information provided about the Delphi procedure. It is not clear how many 'rounds' were conducted.

**Author’s reply:**

We used a modified Delphi technique and explained the procedure more clearly in the paragraphs below. We elaborated on the rationale for the use of the modified Delphi technique, piloting of the informational input, the number of Delphi rounds and the structure of alternating survey rounds and face-to-face meetings, the definition of consensus and standardisation percentages:

(Methods section, first paragraph, page 4-5)
*For the development of this national quality framework for palliative care, we adhered to the Guideline for Guidelines,[2] a complementary tool to the revised international criteria for Appraisal for Guidelines of Research and Evaluation (AGREE II). [3] Considering the broad scope and the multidisciplinary nature of palliative care as well as an extensive amount of stakeholders, we employed a whole-sector approach[4] and we consulted an expert panel in a modified Delphi technique to answer the research question.[5-7]…..*

(Methods section, h. Drafting the quality framework in a modified Delphi procedure, page 8-9)
*To answer the research question, we used a modified Delphi technique among the members of the expert panel. This technique is based on gathering the experts together and discussing the issues from a Delphi survey round in a structured way to reach consensus among all participants simultaneously. Thus, the modified Delphi technique can achieve consensus in a more time- and cost-effective manner.[6]* *We alternated two written Delphi survey rounds with face-to-face meetings of the expert panel.[5-7]* ……

*Data extraction:*

*In the initial face-to-face meeting with the expert panel the selected core documents from the literature review were presented and the format for informational input in the Delphi survey rounds was piloted for clarity and feasibility. For each domain, the research team selected all relevant standards from the core documents, aligned all supporting criteria, and presented them to the expert panel in a first Delphi round (S2 Appendix).* *Each panel member was asked to indicate which of the standards and criteria should be included in the quality framework. Using standardisation percentages, each standard and criterion was graded for admission (> 66% agreed), discussion (50%-66% agreed), or dismissal (<50% agreed).*

*Data synthesis:*

*From the results of the first Delphi round, the research team constructed each domain with the standards and criteria that were accepted or needed to be discussed. Subsequently, the selected standards and criteria were extensively discussed in intermediate face-to-face meetings with all expert panel members and either accepted, discarded, revised, or adapted to the Dutch context. The representatives of patients in the expert panel had the decisive vote whenever a consensus could not be reached.
As a next step, the research team processed the results from the face-to-face meetings and issued a second Delphi round with the expert panel for iterative feedback.*

And

(Methods section, i. Consultation, page 9)
*The research team organised two round-table discussions to inform and involve the steering group and sounding board in the drafting process. Both round-table discussions were followed by written consultation rounds among peers to gather feedback on draft recommendations and assess applicability in clinical practice. These consultation rounds were issued at the same time as the second Delphi round with the expert panel.*

(Methods section, j. Authorisation, page 9)
*After processing feedback from the second Delphi round and the consultation round and obtaining the approval of the final draft of the NQFPC, including its glossary of terms, in a last meeting with the panel members, it was submitted to the associations and organisations represented in the expert panel for final review and authorisation or approval.*

3. Page 13 Table 1 and Line 332 The term 'personal balance' is referred to in the table and in the text. I wonder if this term needs to be defined?

**Author’s reply:**
We understand your request for a clarification of the term and realise the actual meaning of the term may have been lost in translation. We adjusted the term accordingly to “Work-Life balance” in table 1 and clarified the term (Discussion section, page 14 line 351 – 353):

*…education and training of healthcare professionals,[8] that includes effective communication,[9] and learning how to cope with the emotional impact of providing palliative care in order to maintain a healthy work-life balance.[10]*

4. Page 15 Lines 362-363 The authors argue that a strength of their work was building a project team of 'dedicated senior physicians in palliative medicine and medical oncology was critical to facilitate the entire process'. While I can understand that gaining the support of senior clinical leaders is necessary, their statement appears to exclude senior nurses, social workers and allied health professionals. Normally palliative care is delivered by a multidisciplinary team. Perhaps the authors can clarify if health professionals beyond medicine were excluded.

**Author’s reply:**

We can appreciate your concern. The strength we mentioned concerned the constellation of a project /research team of 'dedicated senior physicians in palliative medicine and medical oncology’ in order to gain support of senior clinical leaders. However, to represent the multidisciplinary nature of palliative care the research team consulted stakeholder organisations concerned with nursing, social work etc. and the expert panel that constructed the quality framework comprised healthcare professionals with expertise in anaesthesiology/pain medicine, internal medicine, medical oncology, geriatrics, primary family healthcare, elderly healthcare, nursing, psychology, social care, and spiritual care and were mandated by their national organisations to provide their inputs. We adjusted the manuscript explicitly:

(Methods section, b. Stakeholder involvement, page 5)

*….The research team consulted 26 stakeholder associations and organisations. In line with a whole-sector approach, they represented various medical, nursing and allied health professional disciplines, patients, informal caregivers, volunteers, health insurers, healthcare umbrella organisations, hospice care organisations, and policymakers (S1 Appendix).*

(Methods section, d. Structure for development of NQFPC, page 6)

*….* *This multidisciplinary expert panel represented patients, healthcare providers, and health insurers and was formed to draft the content of the NQFPC. Ten members of the expert panel represented the targeted users of the quality framework and originated from all regions of the Netherlands - various types of hospitals, relevant care settings, and disciplines. They had either generalist or specialist expertise in palliative care in addition to* *expertise in anaesthesiology/pain medicine, internal medicine, medical oncology, geriatrics, primary family healthcare, elderly healthcare, nursing, psychology, social care, and spiritual care* *and were mandated by their national organisations to provide their inputs.*

5. Page 15 Lines 373-374 I realise there is a word limit, but I think the authors undersell the potential transferability of their experience in developing this quality framework.

**Author’s reply:**

Thank you for the compliment. We have taken the liberty to use your introductory observations to adjust the text accordingly:

(Conclusion section, page 16)
*A whole-sector approach using the international AGREE II criteria and a modified Delphi technique to define the content is a feasible, effective, and efficient way to develop a national consensus-based patient-centred quality framework for high-quality palliative care.* *Considering the call to action from the WHO, the process described in this study contains potentially transferable information on how to develop such a framework by taking an inclusive approach and involving stakeholders from civil society rather than regarding palliative care merely as a medical discipline. As such, it may guide other countries’ initiatives to improve the accessibility and availability of palliative care and can contribute to the recognition and integration of palliative care in public health.*

**Reviewer #2:**

Purpose of study clearly stated in the abstract

I commend your mixed generalist and specialist approach

1. Please define the working group? Is that the Delphi participants or the researchers on the project?

**Author’s reply:**

We understand the need for a clarification of the working group and the researchers on the project. Considering the use of a Delphi procedure we adjusted the terms accordingly to research team and expert panel and defined the terms in the manuscript:

(Methods section, a. Research team of senior peers in palliative care, page 5)
*….. a research team of senior peers in palliative care (senior physicians in palliative care and medical oncology) representing the Dutch Society of Professionals in Palliative Care (Palliactief) and the Netherlands Comprehensive Cancer Organisation (IKNL), initiated the development of the Netherlands Quality Framework for Palliative Care (NQFPC). The role of the research team consisted of planning and managing the overall project, consulting and informing the stakeholders and processing the results from the Delphi rounds to inform each next step in the process.*

(Methods section, d. Structure for development of NQFPC, page 6)
*….* *This multidisciplinary expert panel represented patients, healthcare providers, and health insurers and was formed to draft the content of the NQFPC. Ten members of the expert panel represented the targeted users of the quality framework and originated from all regions of the Netherlands - various types of hospitals, relevant care settings, and disciplines. They had either generalist or specialist expertise in palliative care in addition to expertise in anaesthesiology/pain medicine, internal medicine, medical oncology, geriatrics, primary family healthcare, elderly healthcare, nursing, psychology, social care, and spiritual care and were mandated by their national organisations to provide their inputs. Two members of the expert panel were representatives of patients.*

2. What does ‘stakeholder organisations were involved and informed at the management level’ mean? Who is this?

**Author’s reply:**

Stakeholder involvement was described in various sections of the manuscript. For more information on the actual organisations involved, please see S1 Appendix*. Stakeholder associations and organisations involved in development of the Netherlands Quality Framework for Palliative Care.*

To improve readability and better clarify the process we made some adjustments to both the abstract and the manuscript:

(Abstract)

*Six core documents were used in a modified Delphi technique to build the framework with the expert panel, while stakeholder organisations were involved and informed in round-table discussions.*

(Methods section, b. Stakeholder involvement, page 5)
*The research team consulted 26 stakeholder associations and organisations. In line with a whole-sector approach, they represented various medical, nursing and allied health professional disciplines, patients, informal caregivers, volunteers, health insurers, healthcare umbrella organisations, hospice care organisations, and policymakers* *(S1 Appendix). In personal interviews, the research team explored their views on the need for and their willingness to contribute to the development of a national consensus-based quality framework in order to obtain whole-sector support.*

(Methods section, d. Structure for the development of the NQFPC, page 6)
*….., the consulted stakeholder organisations were invited to participate in:
1) a steering group (organisations representing the patient population, the targeted users of the quality framework, and health insurers), or 2) a sounding board (organisations representing patients with specific diagnoses, hospice care organisations, policymakers, research institutes, among others), to support 3) an expert panel for the duration of the development process.*

(Methods section, i. Consultation, page 9)
*The research team organised two round-table discussions to inform and involve the steering group and sounding board in the drafting process. Both round-table discussions were followed by written consultation rounds among peers to gather feedback on draft recommendations and assess applicability in clinical practice.*

3. Your aim is to: develop a national quality framework for improvement of availability and access to high-quality palliative care in a mixed generalist-specialist palliative care model. Your conclusion talks of the Delphi procedure being feasible to develop a framework. This does not answer your aim.

**Author’s reply:**

Thank you. We addressed this accordingly:

(Abstract, purpose)

*We aimed to develop a national quality framework for improvement of availability and access to high-quality palliative care in a mixed generalist-specialist palliative care model. We hypothesised that a whole-sector approach and a modified Delphi technique would be suitable to reach this aim.*

(Abstract, conclusion)

*We successfully developed a national consensus-based patient-centred quality framework for high-quality palliative care in a mixed generalist-specialist palliative care model. A whole-sector approach and a modified Delphi technique are feasible structures to achieve this aim. The process we reported may guide other countries in their initiatives to enhance palliative care.*

(Introduction section, page 4)
*With the intent to improve availability and access to high-quality palliative care for all people with life-threatening illnesses, we developed a national consensus-based quality framework for the optimal organisation and delivery of patient-centred palliative care in a mixed generalist and specialist palliative care model. We hypothesised that a whole-sector approach and a modified Delphi technique could be beneficial for the broad recognition and integration of palliative care.*

(Discussion section, page 14)

*A national quality framework for palliative care seemed an essential step to optimise palliative care for the increasing numbers of patients in a highly fragmented health care system that focuses on cure rather than care. We aimed at a whole-sector approach to obtain broad consent and recognition for high-quality, patient-centred palliative care that could be integrated across health care settings. By inviting patients, healthcare professionals from various medical, nursing and allied healthcare disciplines, health insurers, and policymakers to participate and by building this framework with a modified Delphi technique along the international AGREE II criteria, we combined the aspirations, information, resources, knowledge, and skills of all stakeholders with a scientifically sound structure and reached consensus for the NQFPC that we believe none of the parties concerned could have achieved independently.*

4. Purpose, clear and well written

Have you followed CREDES guidelines for reporting a Delphi? I can’t see it stated or referenced. [https://www.equator-network.org/reporting-guidelines/credes/](https://eur03.safelinks.protection.outlook.com/?url=https%3A%2F%2Fwww.equator-network.org%2Freporting-guidelines%2Fcredes%2F&data=04%7C01%7Calice.firth%40kcl.ac.uk%7C16155006adb7434e3d9808d9b4e90b24%7C8370cf1416f34c16b83c724071654356%7C0%7C0%7C637739735270358516%7CUnknown%7CTWFpbGZsb3d8eyJWIjoiMC4wLjAwMDAiLCJQIjoiV2luMzIiLCJBTiI6Ik1haWwiLCJXVCI6Mn0%3D%7C3000&sdata=P36DcYvEm7ONmvBkv%2BvVzfbJTmqb7XU5Le6YQhAJcho%3D&reserved=0)

**Author’s reply:**

As CREDES guidelines had not been presented at the start of development of our national quality framework in 2015, we adhered to the Guideline for Guidelines.[2] This is a complementary tool to the revised international criteria for Appraisal for Guidelines of Research and Evaluation (AGREE II)[3]. Therefore, instead of CREDES we followed AGREE guidelines for reporting a standard.
(Please see S4 Appendix. AGREE reporting checklist NQFPC).
Part of the AGREE criteria refer to steps used in a modified Delphi technique.
<https://www.equator-network.org/reporting-guidelines/the-agree-reporting-checklist-a-tool-to-improve-reporting-of-clinical-practice-guidelines/>

However, CREDES has been very helpful in improving and clarifying the modified Delphi technique we want to present in the manuscript. We address this further in your next point.

5. I think if you are calling this a Delphi study the Delphi procedure needs to be explained clearly. Also the results from the Delphi need to be clearly stated in tables.

**Author’s reply:**

We used a modified Delphi technique and trust we succeeded in explaining it more clearly in the paragraphs below. However, as this manuscript aims to describe the process of development of the national framework and not the content per se, it is intended as a methodological paper instead of a paper presenting the results from the Delphi study and its separate rounds. Therefore, we included a link in the manuscript (Results section, Finalisation, page 14) to the final result on [www.palliaweb.nl/publicaties/netherlands-quality-framework-for-palliative-care](http://www.palliaweb.nl/publicaties/netherlands-quality-framework-for-palliative-care).

We elaborated on the rationale for the use of the modified Delphi technique, piloting of the informational input, the number of Delphi rounds and the structure of alternating survey rounds and face-to-face meetings, the definition of consensus and standardisation percentages:

(Methods section, first paragraph, page 4-5)
*For the development of this national quality framework for palliative care, we adhered to the Guideline for Guidelines,[2] a complementary tool to the revised international criteria for Appraisal for Guidelines of Research and Evaluation (AGREE II). [3] Considering the broad scope and the multidisciplinary nature of palliative care as well as an extensive amount of stakeholders, we employed a whole-sector approach[4] and we consulted an expert panel in a modified Delphi technique to answer the research question.[5-7]…..*

(Methods section, h. Drafting the quality framework in a modified Delphi procedure, page 8-9)
*To answer the research question, we used a modified Delphi technique among the members of the expert panel. This technique is based on gathering the experts together and discussing the issues from a Delphi survey round in a structured way to reach consensus among all participants simultaneously. Thus, the modified Delphi technique can achieve consensus in a more time- and cost-effective manner.[6]* *We alternated two written Delphi survey rounds with face-to-face meetings of the expert panel.[5-7]* ……

*Data extraction:*

*In the initial face-to-face meeting with the expert panel the selected core documents from the literature review were presented and the format for informational input in the Delphi survey rounds was piloted for clarity and feasibility. For each domain, the research team selected all relevant standards from the core documents, aligned all supporting criteria, and presented them to the expert panel in a first Delphi round (S2 Appendix). Each panel member was asked to indicate which of the standards and criteria should be included in the quality framework. Using standardisation percentages, each standard and criterion was graded for admission (> 66% agreed), discussion (50%-66% agreed), or dismissal (<50% agreed).*

*Data synthesis:*

*From the results of the first Delphi round, the research team constructed each domain with the standards and criteria that were accepted or needed to be discussed. Subsequently, the selected standards and criteria were extensively discussed in intermediate face-to-face meetings with all expert panel members and either accepted, discarded, revised, or adapted to the Dutch context. The representatives of patients in the expert panel had the decisive vote whenever a consensus could not be reached.
As a next step, the research team processed the results from the face-to-face meetings and issued a second Delphi round with the expert panel for iterative feedback.*

And

(Methods section, i. Consultation, page 9)
*The research team organised two round-table discussions to inform and involve the steering group and the sounding board in the drafting process. Both round-table discussions were followed by written consultation rounds among peers to gather feedback on draft recommendations and assess applicability in clinical practice. These consultation rounds were issued at the same time as the second Delphi round with the expert panel.*

(Methods section, j. Authorisation, page 9)
*After processing feedback from the second Delphi round and the consultation round and obtaining the approval of the final draft of the NQFPC, including its glossary of terms, in a last meeting with the panel members, it was submitted to the associations and organisations represented in the expert panel for final review and authorisation or approval.*

6. This study is very interesting, but further development of methods and clearly presented results is needed.

**Author’s reply:**

We trust we have addressed your comments accordingly.

**References**

1.IKNL/Palliactief. Knelpuntenanalyse Palliatieve Zorg in de eerste lijn (Bottleneck analysis of primary palliative care). Utrecht, 2017.

2.Regieraad Kwaliteit van Zorg. Richtlijn voor richtlijnen (Guideline for guidelines). 2012.

3.Brouwers MC, Kho ME, Browman GP, Burgers JS, Cluzeau F, Feder G, et al. AGREE II: advancing guideline development, reporting and evaluation in health care. CMAJ. 2010;182(18):E839-42. Epub 2010/07/07. doi: 10.1503/cmaj.090449. PubMed PMID: 20603348; PubMed Central PMCID: PMCPMC3001530.

4.Virdun C, Luckett T, Davidson PM, Phillips J. Strengthening palliative care in the hospital setting: a codesign study. BMJ Support Palliat Care. 2020. Epub 2020/10/30. doi: 10.1136/bmjspcare-2020-002645. PubMed PMID: 33115832.

5.Junger S, Payne S, Brearley S, Ploenes V, Radbruch L. Consensus building in palliative care: a Europe-wide delphi study on common understandings and conceptual differences. J Pain Symptom Manage. 2012;44(2):192-205. Epub 2012/06/19. doi: 10.1016/j.jpainsymman.2011.09.009. PubMed PMID: 22704058.

6.Ozier W, editor 85-10-10 The Delphi / Modified Delphi Technique : A Consensus Approach to Information Valuation, 1998.

7.Hasson F, Keeney S, McKenna H. Research guidelines for the Delphi survey technique. J Adv Nurs. 2000;32(4):1008-15. Epub 2000/11/30. PubMed PMID: 11095242.

8.de Bruin J, Verhoef MJ, Slaets JPJ, van Bodegom D. End-of-life care in the Dutch medical curricula. Perspect Med Educ. 2018;7(5):325-31. Epub 2018/09/07. doi: 10.1007/s40037-018-0447-4. PubMed PMID: 30187388; PubMed Central PMCID: PMCPMC6191393.

9.Virdun C, Luckett T, Davidson PM, Phillips J. Dying in the hospital setting: A systematic review of quantitative studies identifying the elements of end-of-life care that patients and their families rank as being most important. Palliat Med. 2015;29(9):774-96. Epub 2015/04/30. doi: 10.1177/0269216315583032. PubMed PMID: 25921707; PubMed Central PMCID: PMCPMC4572388.

10.Dijxhoorn AQ, Brom L, van der Linden YM, Leget C, Raijmakers NJ. Healthcare Professionals' Work-Related Stress in Palliative Care: A Cross-Sectional Survey. J Pain Symptom Manage. 2021. Epub 2021/04/18. doi: 10.1016/j.jpainsymman.2021.04.004. PubMed PMID: 33864848.
