# Supplementary material for: Development of a national quality framework for palliative care in a mixed generalist and specialist care model: A whole-sector approach and a modified Delphi technique
Source: PLoS One. 2022 Mar 23;17(3):e0265726. doi: 10.1371/journal.pone.0265726 (PMC8942240; doi:10.1371/journal.pone.0265726)
Supplement: Attachment — Submitted filename: reviewer comments 15.12.21.docx [file pone.0265726.s005.docx]

1. Purpose of study clearly stated in the abstract
2. I commend your mixed generalist and specialist approach
3. Please define the working group? Is that the Delphi participants or the researchers on the project?
4. What does ‘stakeholder organisations were involved and informed at the management level’ mean? Who is this?
5. Your aim is to: develop a national quality framework for improvement of availability and access to high-quality palliative care in a mixed generalist-specialist palliative care model. Your conclusion talks of the Delphi procedure being feasible to develop a framework. This does not answer your aim.
6. Purpose, clear and well written
7. Have you followed CREDES guidelines for reporting a Delphi? I can’t see it stated or referenced. [https://www.equator-network.org/reporting-guidelines/credes/](https://eur03.safelinks.protection.outlook.com/?url=https%3A%2F%2Fwww.equator-network.org%2Freporting-guidelines%2Fcredes%2F&data=04%7C01%7Calice.firth%40kcl.ac.uk%7C16155006adb7434e3d9808d9b4e90b24%7C8370cf1416f34c16b83c724071654356%7C0%7C0%7C637739735270358516%7CUnknown%7CTWFpbGZsb3d8eyJWIjoiMC4wLjAwMDAiLCJQIjoiV2luMzIiLCJBTiI6Ik1haWwiLCJXVCI6Mn0%3D%7C3000&sdata=P36DcYvEm7ONmvBkv%2BvVzfbJTmqb7XU5Le6YQhAJcho%3D&reserved=0)
8. I think if you are calling this a Delphi study the Delphi procedure needs to be explained clearly. Also the results from the Delphi need to be clearly stated in tables.
9. This study is very interesting, but further development of methods and clearly presented results is needed.
